# 390. Corticosteroid Treatment in Severe Patients with SARS-CoV-2 and Chronic HBV Co-infection: A Retrospective Multicenter Study

**DOI:** 10.1093/ofid/ofad500.460

**Published:** 2023-11-27

**Authors:** Sanju Biju, Shon Paulson, Jomel Raju

**Affiliations:** St. Joseph's College of Pharmacy, Thrissur, Kerala, India; St. Joseph's College of Pharmacy, Thrissur, Kerala, India; St. Joseph's College of Pharmacy, Cherthala, Pala, Kerala, India

## Abstract

**Background:**

The impact of corticosteroids on patients with severe coronavirus disease 2019 (COVID-19)/chronic hepatitis B virus (HBV) co-infection is currently unknown. We aimed to investigate the association of corticosteroids with these patients.

**Methods:**

This retrospective multicenter study screened 5447 confirmed COVID-19 patients hospitalized between Jan 1, 2021, to December 1, 2022, in three centers in India, where the prevalence of chronic HBV infection is moderate to high. Severe patients who had chronic HBV and acute SARS-CoV-2 infection were potentially eligible. The diagnosis of chronic HBV infection was based on positive testing for hepatitis B surface antigen (HBsAg) or HBV DNA during hospitalization and a medical history of chronic HBV infection. Severe patients (meeting one of the following criteria: respiratory rate > 30 breaths/min; severe respiratory distress; or SpO_2_ ≤ 93% on room air; or oxygen index < 300 mmHg) with COVID-19/HBV co-infection were identified. The bias of confounding variables on corticosteroid effects was minimized using a multivariable logistic regression model and inverse probability of treatment weighting (IPTW) based on the propensity score.

**Results:**

The prevalence of HBV co-infection in COVID-19 patients was 4.1%. There were 105 patients with severe COVID-19/HBV co-infections (median age 62 years, 57.1% male). Fifty-five patients received corticosteroid treatment and 50 patients did not. In the multivariable analysis, corticosteroid therapy (OR, 6.32, 95% CI 1.17-34.24, P = 0.033) was identified as an independent risk factor for 28-day mortality. With IPTW analysis, corticosteroid treatment was associated with delayed SARS-CoV-2 viral RNA clearance (OR, 2.95, 95% CI 1.63-5.32, P < 0.001), increased risk of 28-day and in-hospital mortality (OR, 4.90, 95% CI 1.68-14.28, P = 0.004; OR, 5.64, 95% CI 1.95-16.30, P = 0.001, respectively), and acute liver injury (OR, 4.50, 95% CI 2.57-7.85, P < 0.001). Methylprednisolone dose per day and cumulative dose in non-survivors were significantly higher than in survivors.

**Conclusion:**

In patients with severe COVID-19/HBV co-infection, corticosteroid treatment may be associated with an increased risk of 28-day and in-hospital mortality.

**Disclosures:**

**All Authors**: No reported disclosures

